# A double-layered fully automated insomnia identification model employing synthetic data generation using MCSA and CTGAN with single-channel EEG signals

**DOI:** 10.1038/s41598-024-74706-9

**Published:** 2024-10-08

**Authors:** Steffi Philip Mulamoottil, T. Vigneswaran

**Affiliations:** School of Electronics Engineering, Vellore Institute of Technology, Chennai, 600127 India

**Keywords:** Augmentation, CTGAN, EEG Signal processing, Insomnia, Sleep disorder, Wavelet transform, Diseases, Health care, Engineering

## Abstract

Insomnia was diagnosed by analyzing sleep stages obtained during polysomnography (PSG) recording. The state-of-the-art insomnia detection models that used physiological signals in PSG were successful in classification. However, the sleep stages of unbalanced data in small-time intervals were fed for classification in previous studies. This can be avoided by analyzing the insomnia detection structure in different frequency bands with artificially generated data from the existing one at the preprocessing and post-processing stages. Hence, the paper proposes a double-layered augmentation model using Modified Conventional Signal Augmentation (MCSA) and a Conditional Tabular Generative Adversarial Network (CTGAN) to generate synthetic signals from raw EEG and synthetic data from extracted features, respectively, in creating training data. The presented work is independent of sleep stage scoring and provides double-layered data protection with the utility of augmentation methods. It is ideally suited for real-time detection using a single-channel EEG provides better mobility and comfort while recording. The work analyzes each augmentation layer’s performance individually, and better accuracy was observed when merging both. It also evaluates the augmentation performance in various frequency bands, which are decomposed using discrete wavelet transform, and observed that the alpha band contributes more to detection. The classification is performed using Decision Tree (DT), Ensembled Bagged Decision Tree (EBDT), Gradient Boosting (GB), Random Forest (RF), and Stacking classifier (SC), attaining the highest classification accuracy of 94% using RF with a greater Area Under Curve (AUC) value of 0.97 compared to the existing works and is best suited for small datasets.

## Introduction

Sleep is an essential physiological phenomenon occurring for the biological restoration of the human body after tiring physical activities^1^. Sleep disorders become apparent for many physical or mental causes and take shape in various versions, such as periodic limb movement disorder, sleep apnea, bruxism, etc^2–4^. The difficulty that arises in normal healthy sleep or irregularity in the sleep pattern results in various types of sleep disorders. Insomnia is one such disorder that occurs due to the occurrence of irregular sleep patterns, mainly noticed in adults^5^. Insomnia is a sleep disorder that occurs due to a person’s inability to fall asleep and stay asleep, which is visible in 30% of the population from the samples taken from different countries^6^. PSG studies performed on insomnia show prolonged sleep latency and increased intervals of awakenings during sleep, indicating a lack of sleep quality^7^. Clinically, insomnia can be identified using subjective and objective means of diagnostic strategy. Subjective means of diagnosis involve using sleep diaries for the computation of sleep evaluation indices, and objective means involve whole-night patient monitoring using polysomnography as per the guidelines released by the American Academy of Sleep Medicine (AASM)^8,9^. Before AASM released the guidelines, they were based on the guidelines present in Rechtschaffen and Kales, which are known as R & K criteria^10^. The consequences of insomnia give way to cardiovascular disease, neurological disease, diabetes, obesity, and many more^11–14^. Insomnia will appear in different faces^15^, such as sleep onset insomnia, sleep maintenance insomnia, early morning awakening insomnia, etc., that can occur due to any health condition or not, any underlying temporary sleep problems that affect mental health. Insomnia will cause a person to suffer for a short period, say a week or two, or can suffer for a long period for more than one month. A chronic variant of insomnia needs treatment and medication that can be diagnosed by suggesting the patient have polysomnography (PSG) recording in the sleep lab, which can be time-consuming, uncomfortable, and barely less effective^16^.

### Related works

The presence of insomnia can be diagnosed effectively by using non-invasive methods such as analyzing the records of biological signals, namely Electroencephalogram (EEG), Electrocardiogram (ECG), Electrooculogram (EOG), and Electromyogram (EMG), or in short, could go for a PSG analysis. However, the clinical approach of sleep study using PSG is not suited for home-based solutions. The conventional method of insomnia detection presented in earlier studies goes with the analysis of sleep stage annotations followed by automated detection. Among sleep disorders, sleep apnea has gained more popularity in the field of research. Even though insomnia appears to be the main factor behind many other lifestyle disorders, it has attained less significance, and limited studies have been available. EEG is a golden tool in studying brain activities, and the single-channel sleep EEG detection model is more comfortable and suitable for home-based environment detection than a multi-channel PSG study in a clinical space. Hence, there is much room for improvement in designing the insomnia detection model, which utilizes EEG signals using a publicly available database.

Earlier studies of insomnia detection utilize spectral power analysis. However, it did not result in automated detection or classification^17^. Later, the successful studies of non-invasive insomnia detection models using machine learning or deep learning algorithms utilize sleep stage analysis^18–21^. Even though EEG signals are largely used in observing brain-related activities in sleep, EOG and EMG signals also show relevance in sleep disorder detection^22^. The performance of classification algorithms relies on the training data fed into them. The training data should be balanced and show negligible redundancy to meet the requirement of the classifier. The main limitation is the deviation in the number of sleep stages utilized for generating data subsets for training. Classifying insomnia signals is challenging without the use of sleep stage analysis. Apart from the usual physiological signals utilized for insomnia detection, actigraphy data can also be used for a classification task that demands a prescribed sleep-in time, which may not result in an accurate diagnosis^23,24^. In addition to analyzing the physiological signals for the insomnia detection models, image processing can also be used. Scalograms of sleep stages of physiological signals, together with machine learning and deep learning models using a wavelet scattering network, resulted in a higher accuracy of 94.9%^25^. Similarly, a complete deep learning network that converts EEG into scalograms produces an accuracy of 98%^26^, which shows another way of insomnia detection models to be more computationally intensive.

The insomnia detection models reviewed were observed to be effective and accurate. However, the main limitation of existing works is the segmentation of raw signals based on sleep stage analysis from sleep annotations performed by trained clinicians during the recording for data enhancement, which may result in data loss to maintain the uniformity of the data subset to train the classification models. However, the sleep stage scoring results in analyzing sleep EEG on a time basis, providing design for the existing models that are reluctant to observe their performance in various frequency bands. The limitations present in previous works can be overcome with an approach independent of sleep stage scoring by analyzing recorded biological signals and extracted features to generate the training data. Hence, in the proposed work availing EEG signal, an approach of data augmentation analyzing performance in frequency bands finds space in generating synthetic data from existing data without changing the underlying characteristics of the signal. The presented model with a single-channel EEG signal applies augmentation on pre- and post-feature extraction to generate the required amount of artificial data that provides data security for the raw data there by refraining from its reconstruction.

The main contributions of the proposed work are:

1. The proposed approach develops a completely automated two-layered augmentation model for insomnia detection utilizing modified conventional signal augmentation (MCSA) and modern deep learning architecture such as conditional tabular generative adversarial networks (CTGAN) for augmentation from single-channel EEG signals without using sleep stage analysis derived from sleep annotations.
2. The approach extracted entropy measures, spectral power, and statistical measures from the unipolar C4-A1 channel. The work examined the effect of each augmentation layer in different frequency bands and a combination of both augmentation layers using various machine learning models.
3. It also investigated the behavior of each frequency band in insomnia detection with the involvement of augmentation methods.
4. The model attains a greater accuracy of 94%, as obtained for the random forest machine learning model with an Area Under Curve (AUC) of 0.97, a sensitivity of 95%, and a classification error rate of 0.06.

The rest of the paper is detailed as follows. Section 2 shows the data used by the proposed work. Section 3 explains the background of the study, and Sect. 4 elaborates on the proposed methodology. Section 5 briefs the classification and performance evaluation used for the analysis of the study, followed by results and discussion in Sects. 6 and 7 and conclusion in Sect. 8.

## Data

The proposed method aims to analyze EEG sleep signals for the development of the classification model. The work makes use of the CAP sleep database, which can be downloaded from the publicly available Physionet website^27^. The dataset comprises the whole night sleep recording of insomnia subjects as well as healthy subjects. There were 25 subject’s polysomnography recordings available in the dataset; from that, 16 were healthy, and 9 were insomnia sufferers. The sampling frequency of the recorded datasets was not uniform and varied as 100,128,200 and 512 Hz. The work focused on the selection of subjects with a uniform sampling frequency. Hence, the proposed work has chosen the subjects with a sampling frequency of 512 Hz. Polysomnography, a standard tool that records sleep architecture^28^, uses a globally accepted 10–20 electrode system for the recording of the subjects. Moreover, polysomnography recordings provide the recordings of other physiological signals, such as an electroencephalogram (EEG), which will provide brain activity during sleep. An electrocardiogram (ECG) shows heart rate variations while sleeping. Electrooculogram (EOG) and Electromyogram (EMG) provide eye activities and muscle movements during sleep. All the signals were found relevant for the identification of insomnia during sleep. Our analysis focused on single EEG signals instead of multi-channel in generating the input features for the training data of the machine learning model^29^. On focusing the real-time analysis in the identification of insomnia, single-channel signals are preferred over multi-channel^30^ for getting a comfortable sleep environment and avoiding the “first night effect” in recording the signal, enhancing the accurate diagnosis of insomnia even in a home environment. In this work, we used the C4-A1 channel EEG signal with a sampling frequency of 512 Hz. The details of the dataset utilized for the work and the inferences from the dataset are described in Table 1.

**Table 1:** Details of the CAP dataset used in the study.

Sub	Gen	Age	sleep start	sleep end	in bed duration	time in bed(min)	SO	W after SO	TST (min)	SL (min)
INS2	F	58	18:25:37	8:22:38	13:57:01	837.02	23:11:38	266	551	286.02
INS4	F	58	21:34:04	3:40:04	6:06:00	366	21:47:34	35	352.5	13.5
INS5	F	59	17:58:48	8:18:18	14:19:30	859.5	23:30:18	256	528	331.5
INS6	F	54	22:37:17	7:25:17	8:48:00	528	22:56:47	456	460	19.5
INS7	F	47	19:58:14	8:19:14	12:21:00	741	21:54:44	377	624.5	116.5
INS8	M	64	22:43:04	5:42:34	6:59:30	419.5	23:14:34	168	388	31.5
n1	F	37	22:09:33	7:42:33	9:33:00	573	22:15:03	29	567.5	5.5
n2	M	34	22:19:06	6:38:36	8:19:30	499.5	22:23:06	136	495.5	4
n3	F	35	23:06:12	7:26:12	8:20:00	500	23:07:12	135	499	1
n5	F	35	22:49:48	7:13:18	8:23:30	503.5	22:51:48	7	501.5	2
n10	M	23	23:24:52	6:34:22	7:09:30	429.5	23:56:22	4	398	31.5
n11	F	28	22:37:16	7:23:16	8:46:00	573	22:15:03	21	508	18

Table 1 describes the details of the subject with the same sampling frequency, showing gender, age, and sleep duration available on the Physionet website, as well as the inferences we developed from the dataset. The table clearly shows that the disorder more often affects females than men and details each participant’s sleep duration, sleep onset, wake after sleep onset, and sleep latency; the table indicates INS for insomniac subjects, and n shows normal subjects with an age above 46 years. The standard amount of sleep latency (SL) shows how long it takes for a person to enter sleep^31^, which has to be less than 20 min for healthy sleep, which can be proven by the inference shown in Table 1. Sleep onset (SO) indicates the time it takes for a subject to enter into sleep. TST indicates total sleep duration that can be obtained by taking the difference between SL and total time in bed.

## Background

In a human sleep, there can be a sudden change every 30 s to a different sleep state that a manual interpretation can’t be faultless for longer hours, say a whole night. Hence, there might be chances of mistakes while performing sleep scoring. Insomnia is clinically diagnosed by keeping the subject to sleep in a clinical environment with a trained technician to note down the sleep stages manually. Also, analyzing sleep on time segmentation is extremely high in the number of epochs, and the same signal characteristics going to be repeated multiple times, which will be challenging to interpret. The main contribution of the proposed work is to abstain from using sleep stage scoring for the analysis of the work, thereby framing the model extensively automated in all stages of the study, hence avoiding the involvement of the human hypothesis. The model cannot be considered a diagnostic tool but can be utilized as an initial perception regarding the disorder by keeping away the sleep discomforts experienced from sleep labs. In the proposed work, the study pays attention to analyzing the frequency bands (FBs) of EEG signals. The EEG signal frequency is spread in 0.5–45 Hz and is available in various frequency bands. The subsequent analysis of each frequency band gives way to a better examination regarding the detection of insomnia without using sleep scoring. The EEG frequency bands and their frequency range are dictated in Table 2^32,33^. The sleep state of a person during each frequency band is also mentioned, and it is observed from the table that a deep sleep state is visible in the delta frequency, and the sleep state changes corresponding to the occurrence of each frequency band. In building insomnia detection models, we will mainly create subsets that consider these frequencies for analysis.

**Table 2:** EEG frequency bands and frequency range.

Range of EEG Frequency (Hz)	Frequency band	Description
0.5-4	Delta	Deep sleep
4–8	Theta	Deeply relaxed, inward-focused
8–12	Alpha	Very relaxed, passive attention
12–30	Beta	Anxiety dominant, active external attention, relaxed
> 30	Gamma	concentration

The samples are subjected to a frequency decomposition of up to 5 frequency bands using wavelet decomposition. Earlier methods used the Fourier transform for frequency decomposition; later, Daubechies Wavelets were chosen for frequency decomposition, allowing us to analyze the signal in time and the frequency domain. Daubechies wavelet is a short-duration waveform of discrete wavelet transform that decomposes the given signal into several sets^34,35^. Since the frequency of the subject’s sleep will vary every 30 s to a different frequency corresponding to the sleep stage, it is necessary to analyze each sleep transition abruptly. Daubechies wavelet is the suitable choice for analyzing signals with sharp transitions and is also best suited for the non-stationarity of EEG signals.

## Proposed methodology

**Fig. 1:**
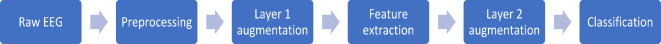
Workflow of the proposed method.

The proposed approach consists of 5 steps in developing the automatic detection model of insomnia. The approach uses augmentation in creating the dataset instead of sleep stage classification through sleep scoring. The workflow of the proposed approach is shown in Fig. 1. The model comprises preprocessing, signal level augmentation (Layer 1), feature extraction, feature level augmentation (Layer 2), and classification. Preprocessing is performed in two stages as the raw EEG signal is initially denoised and filtered using a Butterworth bandpass filter with a frequency range of 0.5–45 Hz. The model comprises two layers of augmentation in analyzing the hidden minute characteristics present in the signal. Augmentation should be performed at the signal level as well as at the feature level. The work uses traditional methods of signal augmentation for signal level augmentation, whereas for feature level augmentation, it uses modern deep learning techniques.

### Preprocessing

**Fig. 2:**
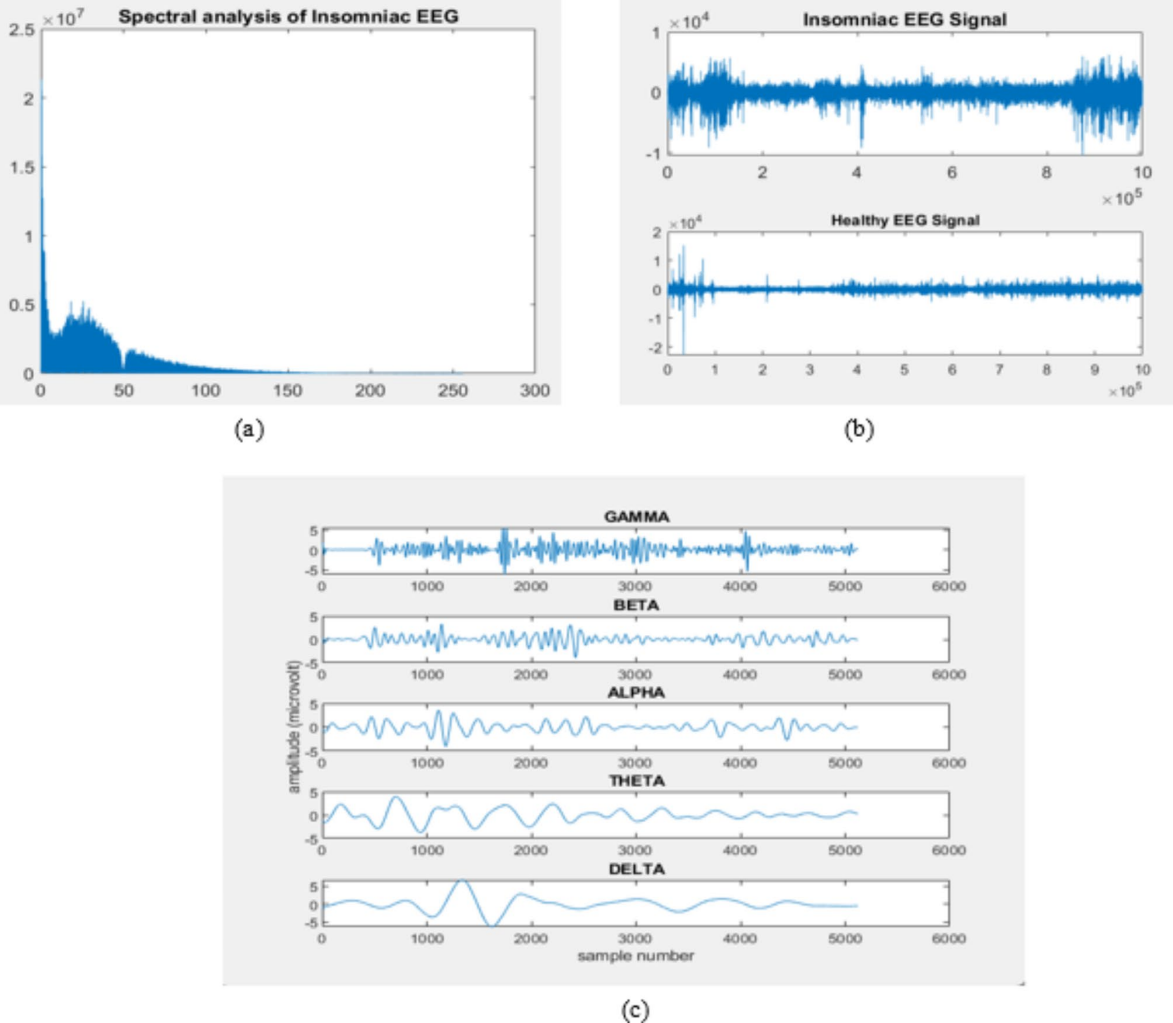
(**a**) spectral analysis of insomniac EEG, (**b**) insomniac and healthy EEG signal, and (**c**) decomposed frequency bands of the sample healthy subjects.

The initial stage of EEG analysis is to process the signal according to the requirement for developing a classification model for an accurate analysis. The pre-processing step comprises three levels of operation, including denoising, filtering, and frequency decomposition. The EEG signal is prone to noise at the time of recording. The possibility of artifacts present in real-time signals is baseline drift, powerline interference, etc. As we are utilizing a publicly available dataset, it is not necessary to perform very efficient signal-denoising techniques suitable for each artifact. The work uses a wavelet denoising technique for artifact removal. The denoised signal is filtered for 0.5–45 Hz using a Butterworth bandpass filter. The filtered signal is subjected to a frequency decomposition of 5 frequency bands. The sampling frequency utilized here is 512 Hz, and hence, a DB8 wavelet is leveraged in the analysis. It generates approximate level coefficients and Detail coefficients by using low-pass and high-pass filters present in the structure. Figure 2 shows the EEG waveform of healthy and insomniac subjects and the frequency spectrum of insomnia subjects, with its decomposed frequency bands. The frequency distribution of the EEG signal in Fig. 2(a) indicates that the signal is distributed over 0–50 Hz. Therefore, the EEG signal can be filtered around the depicted frequency range. It also shows the 5 frequency bands of healthy sleep patterns, namely alpha, beta, gamma, theta, and delta. The decomposed EEG frequency bands explicitly express the sleep pattern in each sleep stage, which assists us in extracting the hidden characteristics present in the signal using suitable feature extraction methods. To analyze the EEG signal frequency bands, it is essential to examine the signal in its multiple forms. Signal scaling and noise injection provide a way to analyze the EEG signal frequency bands without giving an abrupt change in their behavior.

### Synthetic data generation using MCSA

**Fig. 3:**
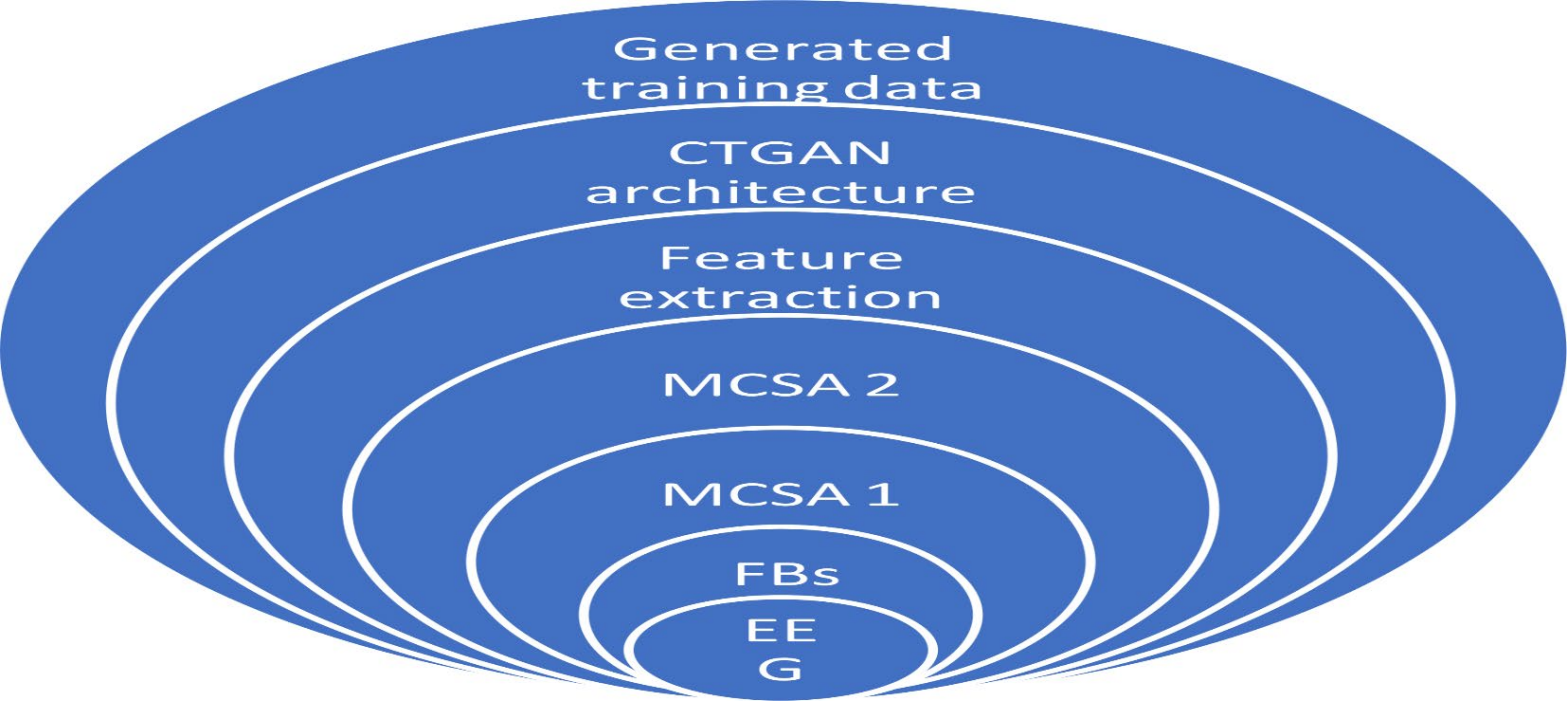
Generation of training data using two-layered DA to the machine learning model.

The process of transforming the input sample to a new one without changing the behavior of the input data is termed data augmentation. Data augmentation (DA) is commonly used for image processing^36^. In the case of selecting a suitable method for augmenting the EEG signal for insomnia diagnosis is a difficult task due to the nonstationary and nonlinear properties of the signal. The model performance is based on the selection of the DA methods. Two ways we can perform DA in signal processing applications, one in generating new signal samples from existing ones and the other in generating new data from the extracted features. The proposed approach for generating a data set using a two-layered DA, as shown in Fig. 3, adopted two DA techniques to develop the model. The 1st level of augmentation is performed together by MCSA-1 and MCSA-2, in which both will generate artificial signals from raw EEG by performing augmentation considering small perturbations on the raw data. Then, the stage of feature extraction is carried on the artificially generated signals from the 1st augmentation layer. The 2nd level of augmentation is implemented using a modified form of Conditional GAN architecture (CGAN) called Conditional tabular GAN (CTGAN), which is designed for synthetic tabular data generation. Feature level augmentation is enough to generate immense data to train the machine learning model because we are using a modified Generative adversarial network (GAN) to duplicate the input data. The architecture will generate as many numbers of data as given in the algorithm. However, it will fail to consider various forms of the signal; instead, it will simply fake the given input data. In contrast, MCSA methods provide a way to create new samples by analyzing different forms of the signal in different frequency bands.

### Overview of layer 1 augmentation

**Fig. 4:**
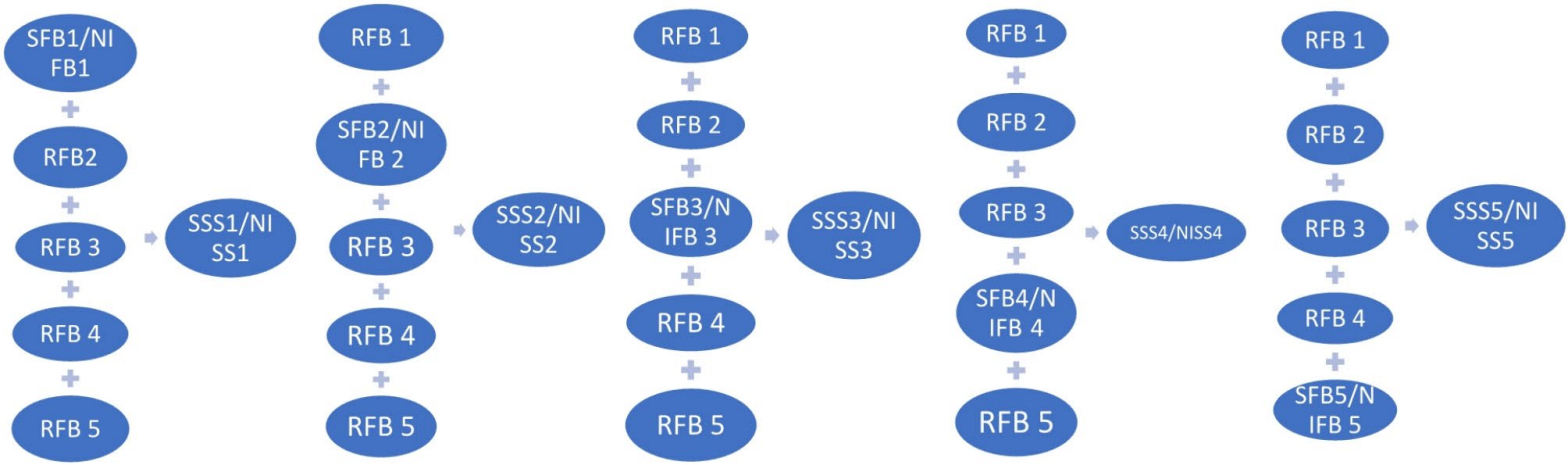
Layer 1 augmentation of the synthetic signal generation.

The decomposed EEG frequency bands are subjected to layer 1 augmentation using MCSA methods. Augmentation of signals is a technique that generates new signals from existing ones. Many conventional and modern signal augmentation methods are available. Signal scaling, noise injection, signal shifting, and time reversing are a few traditional signal augmentation methods^37^. In contrast, modern deep learning techniques such as Generative Adversarial Networks (GAN), Conditional GAN (CGAN), and tabular GAN (TGAN) are some of them. GANs are utilized to generate synthetic data in case of data scarcity problems. All the machine learning and deep models used for automated detection and classification require a good amount of training data to accurately predict performance. The data scarcity issue can be rectified by employing synthetic data generation^38,39^. Synthetic data can be generated at the signal level and the feature level. Signal-level synthetic data generation can be accomplished with the involvement of traditional augmentation methods, and feature-level synthetic data generation can be accomplished using modern deep learning techniques. The selection of a data augmentation method suitable for improving the performance of the model is cumbersome. The selection of a suitable method is based on the properties of the input data. Our input is an EEG signal, and its nonlinearity and nonstationary property allow us to select noise addition as a suitable method to develop the data augmentation model. Here, we used white Gaussian noise to add to each frequency band. Another suitable method is scaling the signal in an elevated manner or in a decremented way in its amplitude. The attenuation of a signal gives a chance of reducing the signal strength and, hence, information loss; the work has chosen signal amplification suited for the model. Thus, the proposed work depicted in Fig. 4 shows layer 1 EEG signal augmentation, which uses signal scaling and noise injection for the generation of Scaled Synthetic Signals (SSS) and Noise Injected Synthetic Signals (NISS) from the existing samples. The process can be illustrated as follows: the raw signal is arranged as a group of segments, where each segment constitutes different frequency bands, and augmentation will be performed beginning from the 1st segment, and it gets combined by being added to the remaining raw FBs, and then the augmentation shifted to the second segment keeping all the other raw FBs added to the augmented second segment and the process continued till it reaches the last segment. After performing augmentation in each frequency band in each segment, a new synthetic signal is produced, avoiding much deviation from the characteristics of raw data.

The first level of augmentation before feature extraction is implemented in two cycles, MCSA-1 and MCSA-2, where the first cycle of synthetic signal generation, MCSA-1, is done by using signal scaling, and the second cycle of signal generation, MCSA-2, is done using noise injection. MCSA-1 generates a set of SSS from 1 SFB and 4 RFBs in which each signal has a scaled segment of frequency bands. The generated signals of each scaled frequency band (SFB) are convoluted with filtered raw EEG signals, producing another signal set from the existing one. Hence, signal addition and convolution operation generate two sets of new EEG signals in MCSA-1, resembling the raw signals with an elevated or decremented amplitude.

On the other hand, in MCSA-2, the second cycle of layer 1 augmentation, the decomposed frequency bands in each segment are injected with white Gaussian noise in every step. The noise-injected frequency band in each segment is then added to the remaining RFBs and produces a new synthetic signal from the existing one. The process of noise injection and addition continues to the rest of the frequency bands to increase the signal samples. The generated noise-injected samples are convoluted with filtered raw EEG signals, producing new signals. Augmentation proceeds for all subjects, including insomniac sufferers as well as healthy subjects. MCSA-1 and MCSA-2 generate 457 artificial signals from the existing raw EEG through conventional signal augmentation. Signal analysis implies investigating the hidden characteristics present in the signal by extracting the features representing the signal samples. Figure 4 depicts the layer 1 augmentation using the MCSA operation performed in both cycles MCSA-1 & MCSA-2.

### Feature extraction

The process of extracting features is one of the main stages in determining the performance of the model and is considered a vital process in biomedical signal processing. We can extract linear, nonlinear, or spectral characteristics in signal processing to represent the dataset. The extracted features are used to train machine-learning and deep-learning models. Non-linear feature extraction was found significant in the EEG signal analysis due to the nonlinearity of the EEG signal. The work extracts a total of 9 features, including entropy measures say sample entropy, spectral entropy, Shannon entropy^40–42^, log energy entropy, spectral band power, and statistical measures such as mean, standard deviation, skewness, and kurtosis. Entropy features are an efficient feature that is better suited to give the hidden information present in the EEG signal. Spectral power varies for each frequency band, and the extracted band power values show the spectral power of each frequency band. Entropy measures are very significant in EEG analysis to examine the electrical activity of the brain and nonlinear interactions between the neurons in transmitting signals. Entropy measures are greatly used in the early diagnosis of the irregular pattern in the brain signals with the occurrence of sleep-related disorders.

**Fig. 5:**
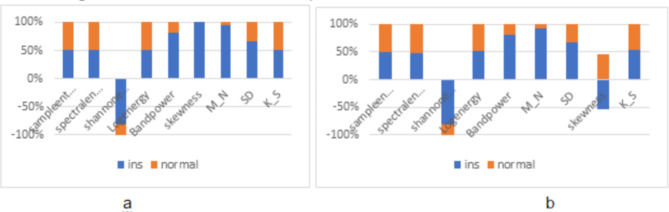
Comparison of extracted features for insomnia and healthy subjects. (**a**) after scaling, (**b**) after noise injection.

Figure 5 shows the distribution of each extracted feature from the generated data after layer 1 augmentation for insomniac and normal subjects. The features extracted after scaling of each frequency band vary for healthy as well as insomniacs, which can be well explained in Fig. 5a), and features extracted after noise injection in each frequency band vary as indicated in Fig. 5b). It can be shown that an immense variation can be observed after scaling in healthy and insomniacs for Shannon entropy, band power, and statistical features such as skewness, mean, and standard deviation. In contrast, a slight variation is visible for the rest of the features, say sample entropy, spectral entropy, log energy, and kurtosis. In the case of noise injection, sample entropy, spectral entropy, log energy, skewness, and kurtosis differ in small amounts. Both augmentation techniques of signal scaling and noise injection generate synthetic EEG signals from the recorded samples. The extracted features increase the sample size for the training data in building a superior machine learning model to perform classification.

### Overview of layer 2 augmentation

After the creation of synthetic signal samples in layer 1 followed by feature extraction, layer 2 augmentation is performed using GAN architecture^43,44^. Layer 2 augmentation was done using Conditional Tabular Generative Adversarial Networks (CTGAN). CTGAN is a method for generating synthetic tabular data of rows from the given distribution^45,46^. GAN-based synthetic data generation is usually performed for image processing but in signal processing, we have to generate synthetic data either by producing new signal samples or by duplicating the extracted features. The proposed approach of biomedical signal processing for insomnia detection performs augmentation techniques in creating new signal samples together with new features to develop the training data for the classification model. The main advantage of tabular data generation is ensuring the security of real-time data. The sleep pattern is much more secure data and is not to be disclosed in the case of higher personals, and hence, this approach is very much suited.

**Fig. 6:**
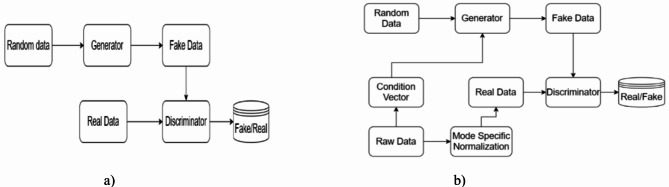
(**a**) Basic GAN architecture (**b**) CTGAN architecture.

A generative model termed GAN is composed of two blocks, mainly a generator and a discriminator. The generator network works toward generating new data samples from the real ones given to the network. The discriminator functions as a network that discriminates the generated data as fake or real. GAN is an adversarial training model that generates realistic data at the end of the training. Different types of GAN architecture are available, such as Vanilla GAN, Wasserstein GAN (WGAN), Conditional GAN (CGAN), and Conditional Tabular GAN (CTGAN). The proposed approach requires a GAN architecture that should generate tabular data considering categorical variables. CTGAN, a modified GAN architecture, seems a good choice for the appeal required by the proposed model. Figure 6a) shows the basic structure of GAN architecture. In GAN architecture, a block of condition vector and mode-specific normalization is not seen, which can be seen in Fig. 6b), which shows the architecture of CTGAN. The generator in both structures generates fake data resembling the distribution present in the real data given as input. The task of the discriminator is to discriminate the data generated as fake/real. The generator generates the data till it can fool the discriminator. The main advantage of CTGAN is the realization of categorical variables in the synthetic data generation, which is perfectly suited to the proposed approach of insomnia detection. CTGAN is widely used in applications where there is a sufficiency in the modeling of tabular data that is visible in mixed formats of both quantitative and qualitative data. CTGAN is intended for operating mixed tabular data chores in mode-specific normalization and conditional training approaches^47^.

**Fig. 7:**
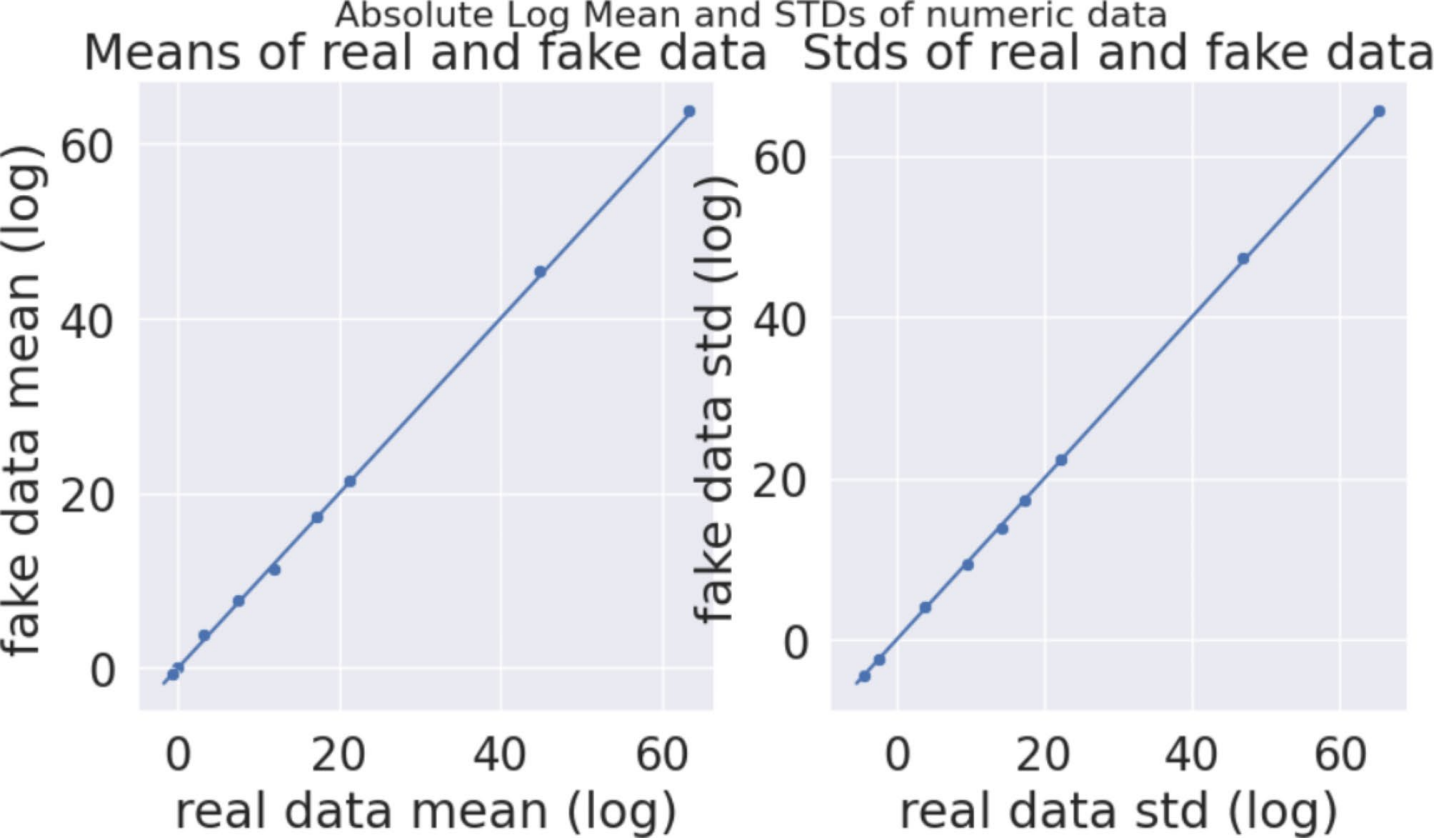
Mean and standard deviation of real and fake data.

Mode-specific normalization considers the floating values that behave in a non-Gaussian distribution manner, which is present in the given input data, whereas a conditional vector identifies the categorical variables. CTGAN resolves the problem of data generation by encoding every single column of tabular entries and categorical variables into condition vectors. These condition vectors are sampled and fed as input to the Generator block. The extracted features from the augmented signals were fed into CTGAN architecture in the proposed model for the next level of augmentation. They were trained to generate synthetic tabular rows and columns data from the extracted layer 1 features. It is hyper-tuned to 2000 epochs with a sample size 10,000 to obtain a distribution similar to the actual data fed into the architecture. Hence, both augmentation layers generated artificial data from the existing one without changing the real data’s underlying distribution, as shown in Fig. 7. The generated training data from the layer 2 augmentation is given to the classifier algorithm.

## Classification and performance evaluation

The generated synthetic data from two-layered data augmentation models are given to machine learning models for classification. The behavior of the dataset influences the choice of the classification model. Since the dataset is smaller in number, machine-learning (ML) models are preferred over deep-learning (DL) models. The data generated by the proposed approach is artificial; hence, there can be a probability of overfitting issues emerging in the classification task. Significance must be given when selecting a machine-learning model free from overfitting issues. The proposed work utilizes a Decision Tree (DT), Ensemble Bagging Decision Tree (EBDT), Gradient Boosting (GB), Random Forest classifier (RF), and Stacking Classifier (SC) for training the augmented data. The hyperparameters used in the machine learning models that enhance the accuracy of the training data are described in Table 3. The parameter of the random_ state defines the shuffling of data before applying the split. Random_state for an integer value produces the same result in iterations. The default criterion used in the decision tree model is unsuitable for evaluating each node’s impurity; hence, the model chose entropy as the criterion. Entropy features, spectral band power, and statistical features are extracted from decomposed augmented frequency bands. The performance evaluation of the classification models is executed by leveraging parameters like accuracy, sensitivity, specificity, F1 score, precision, recall, and classification error rate. A greater classification accuracy of 94% is obtained for the RF ML model with a sensitivity of 95%.

**Table 3:** Machine learning models and hyperparameters.

Machine learning models	Hyperparameter
Decision tree	Random_state = 22, max_depth = 10, criterion = entropy
Ensembled Bagged Decision Tree	Random_state = 22, n-estimator = 100, base_estimator = DecisionTreeClassifier() criterion=”entropy”
Gradient Boosting	Random_state = 22, n_estimator = 100, learning rate = 0.1
Random Forest	Random_state = 22, n_estimator = 100
Stacking Classifier	Layer1 = EBDT, RF, DT, Meta_classifier = GB

## Results

The synthetic data generated from the CTGAN architecture should preserve the structure and statistical properties of real data and must be validated to identify its similarity to real data. High-quality training data can only produce higher accuracy on machine learning models. The diagonal dots in Fig. 7 show the mean and standard deviation of the real and fake data, which is of good quality. The quality of synthetic data increases as the number of epochs increases while training the model. CTGAN produces numerical synthetic data similar to the columns in real data, which can be categorical, continuous, or discrete. Meanwhile, the correlation matrix used for validation shows the correlation between the variables that cannot be varied while generating synthetic data. The correlation coefficient in every cell shows the solid/weak relationship between the variables, which can be useful in building the classification model. The proposed model uses 6 insomniacs and 6 healthy subjects with a uniform sampling frequency of 512 Hz to develop the model. The work has been carried out by analyzing a performance evaluation of sub-datasets comprising all 5 decomposed frequency bands for every subject and the combined frequency bands. The evaluation provides the model performance among various frequency bands that helps in the computation of the selection of frequency bands suited for detection. The sleep state in every human varies in alpha, beta, delta, and theta frequency bands. The model exhibits a greater performance in all 4 frequency bands, and since gamma frequency did not contribute to the sleep state, it shows less accuracy in detection. RF shows a higher accuracy when considering the sub-data set with the features extracted from all frequency bands that constitute the subject’s sleep for the whole night.

**Fig. 8:**
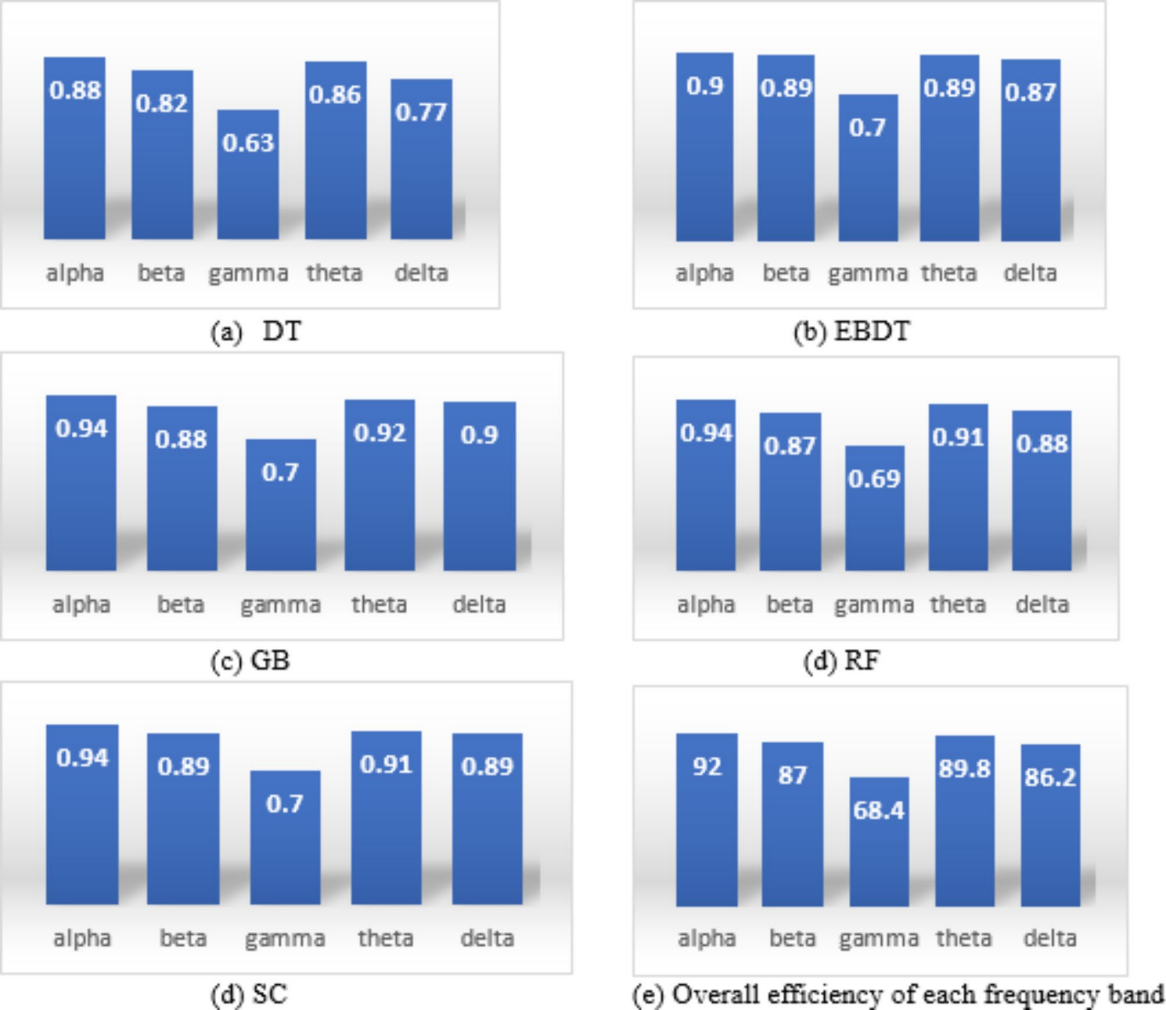
Performance of EEG frequency bands for combined datasets.

**Fig. 9:**
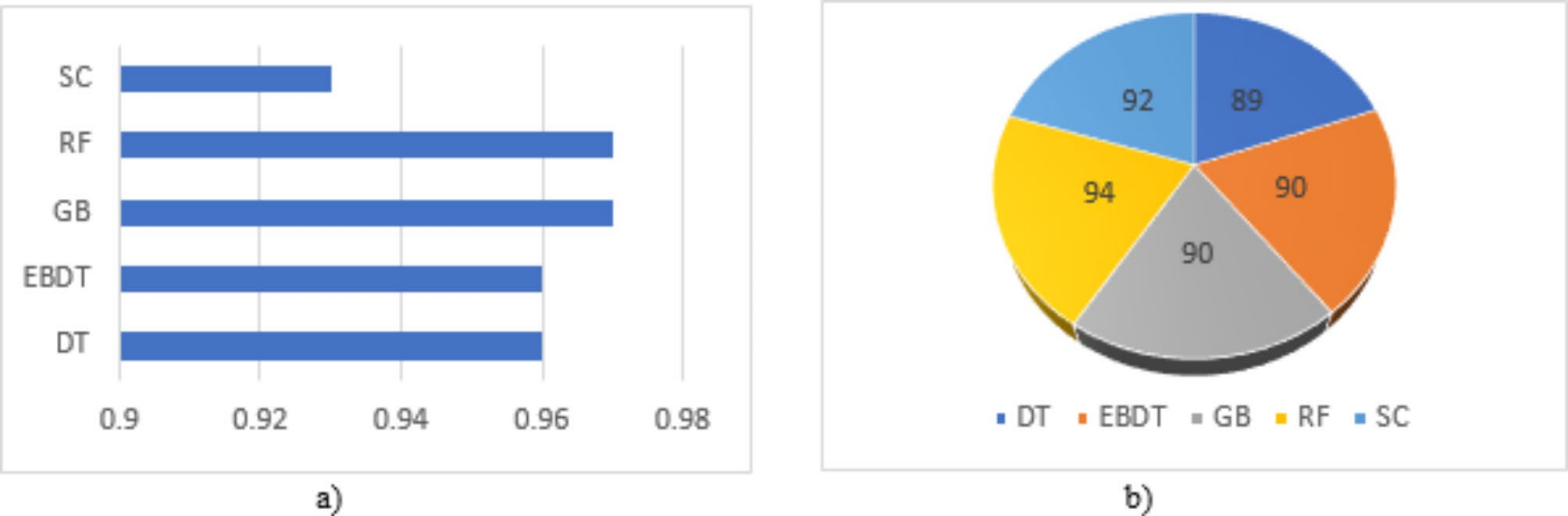
(**a**) AUC and (**b**) Accuracy values of combined data on different ML models.

It is seen from feature extraction that a small variation is visible in the features extracted from all 5 frequency bands, and compared to all other machine learning models used in the model, RF exhibits a greater performance in identifying these minor variations and also shows a minimum CER. The model also validates the result by using a 5-fold cross-validation model. The model uses 75% of training data and 25% of testing data for classification. Figure 8 shows the accuracy of EEG frequency bands while evaluating the performance of each frequency band in the identification of insomnia in different machine learning models, and Fig. 9 shows Area Under Curve (AUC) values and accuracy for the different classification models for the combined frequency bands sub-dataset. Figure 8 expresses the relevance of EEG frequency bands in identifying the disorder. It exhibits the accuracy values of different frequencies in various machine-learning models. On average, it is observed that the alpha band exhibits the highest performance, followed by theta bands, which constitute excellent efficiency in detecting insomnia. The AUC values presented in Fig. 9a) show the ability of a model to distinguish between the classes. The more AUC value there was, the better the model performed. In the presented approach, the AUC values for all the ML models are higher. Both figures show that RF and GB show the highest AUC values compared to other ML models, and RF shows greater accuracy.

### Evaluation metrics

Tables 4, 5, 6, 7, 8 and 9 represents the model performance in all frequency bands. The performance metrics for individual and combined frequency bands in various machine-learning models are shown in Tables 4, 5, 6, 7, 8 and 9. The highlighted values in the table in each category show the highest measure evaluated for each metric. The table shows that while analyzing each frequency band, the gamma frequency band shows very little accuracy in performance, which can be omitted in later studies regarding sleep analysis.

**Table 4:** Evaluation metrics for combined frequency band.

ML models	Accuracy	Sensitivity	Specificity	Precision	Recall	F1score	AUC	CER
DT	0.89	0.93	0.86	0.86	0.93	0.89	0.96	0.10
EBDT	0.91	0.92	0.90	0.91	0.92	0.91	0.96	0.09
GB	0.90	0.93	0.91	0.92	0.90	0.91	**0.97**	0.096
RF	**0.97**	**0.95**	**0.93**	**0.93**	**0.95**	**0.94**	**0.97**	**0.06**
SC	0.92	0.94	0.90	0.91	0.94	0.93	0.93	0.07

**Table 5:** Evaluation metrics for alpha frequency band.

ML models	Accuracy	Sensitivity	Specificity	Precision	Recall	F1score	AUC	CER
DT	0.88	0.87	0.89	0.91	0.87	0.89	0.88	0.12
EBDT	0.90	0.86	0.91	0.93	0.89	0.91	0.97	0.1
GB	**0.94**	0.94	0.94	**0.95**	0.94	**0.94**	0.97	**0.06**
RF	**0.94**	0.93	**0.95**	**0.95**	0.93	**0.94**	**0.98**	**0.06**
SC	**0.94**	**0.95**	0.92	0.92	**0.95**	**0.94**	0.94	**0.06**

**Table 6:** Evaluation metrics for beta frequency band.

ML models	Accuracy	Sensitivity	Specificity	Precision	Recall	F1score	AUC	CER
DT	0.82	0.81	0.82	0.80	0.81	0.73	0.81	0.18
EBDT	**0.89**	**0.90**	**0.89**	**0.86**	**0.90**	**0.88**	**0.96**	**0.11**
GB	0.88	0.89	0.87	0.83	0.89	0.86	**0.96**	0.12
RF	0.87	0.87	0.87	0.83	0.87	0.85	**0.96**	0.20
SC	**0.89**	0.89	0.88	0.85	0.89	0.87	0.88	**0.112**

**Table 7:** Evaluation metrics for Gamma frequency band.

ML models	Accuracy	Sensitivity	Specificity	Precision	Recall	F1score	AUC	CER
DT	0.63	0.62	0.65	0.62	0.62	0.62	0.63	0.36
EBDT	**0.70**	0.68	**0.73**	**0.72**	0.68	**0.70**	0.77	0.36
GB	**0.70**	**0.70**	**0.71**	0.67	**0.70**	0.69	**0.78**	0.36
RF	0.69	0.68	0.69	0.65	0.68	0.66	0.77	**0.31**
SC	**0.70**	**0.70**	**0.71**	0.67	**0.70**	0.69	0.70	0.36

**Table 8:** Evaluation metrics for Theta frequency band.

ML models	Accuracy	Sensitivity	Specificity	Precision	Recall	F1score	AUC	CER
DT	0.86	0.88	0.84	0.85	0.88	0.86	0.86	0.14
EBDT	0.89	0.88	0.90	0.90	0.88	0.89	0.96	0.10
GB	**0.92**	**0.92**	**0.93**	**0.93**	**0.92**	**0.93**	**0.98**	**0.07**
RF	0.91	0.90	**0.93**	**0.93**	0.90	0.91	0.97	0.08
SC	0.91	0.90	0.92	0.92	0.90	0.91	0.91	0.08

**Table 9:** Evaluation metrics for delta frequency band.

ML models	Accuracy	Sensitivity	Specificity	Precision	Recall	F1score	AUC	CER
DT	0.77	0.92	0.61	0.72	0.92	0.81	0.85	0.22
EBDT	0.87	0.93	0.80	0.83	0.93	0.88	0.95	0.13
GB	**0.90**	**0.95**	0.85	0.87	**0.95**	**0.91**	**0.97**	**0.1**
RF	0.88	0.92	0.84	0.86	0.92	0.89	0.96	0.12
SC	0.89	0.92	**0.87**	**0.88**	0.92	0.90	0.89	**0.10**

**Fig. 10:**
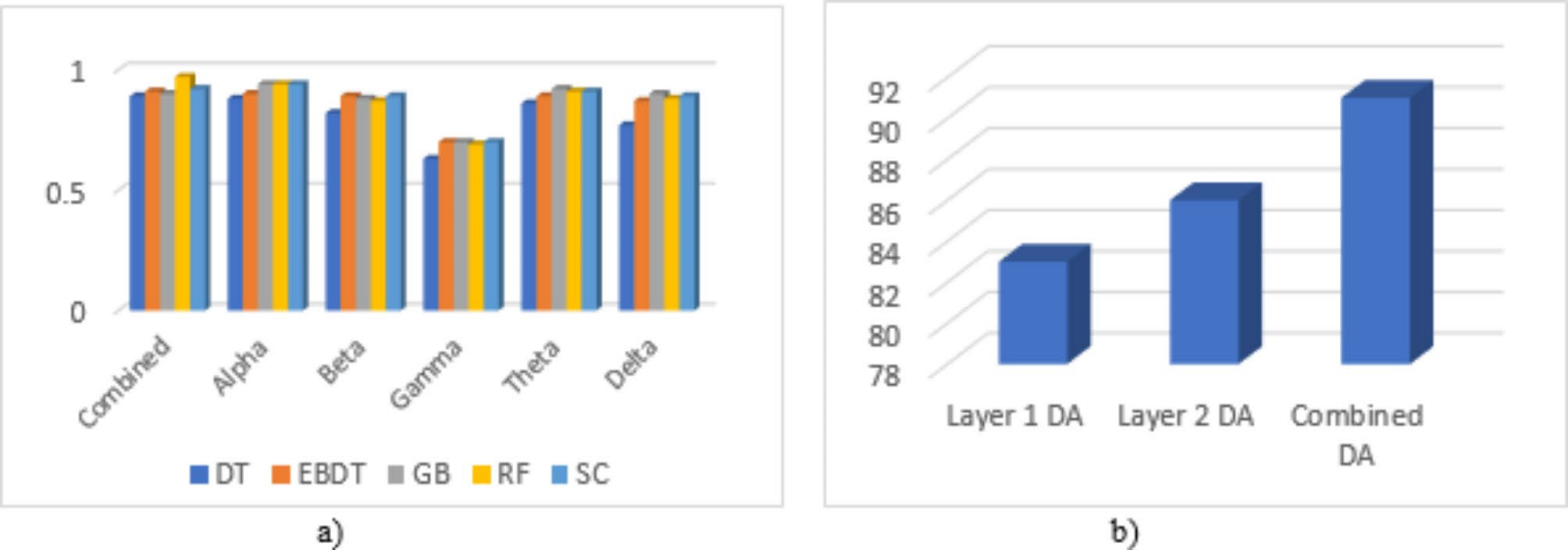
(**a**) Comparison of accuracy of ML models for various frequency bands (**b**) Comparison of accuracy of various DA methods using EBDT.

Observing the performance parameters of each model in various frequency bands shown in Tables 4, 5, 6, 7, 8 and 9 proves that finding the best model suited for the automatic identification of insomnia is cumbersome. On the contrary, it isn’t easy to find which frequency band gives the most accurate detection. The graphical representation of model performance in terms of accuracy is shown in Fig. 10a). The models are evaluated for a data subset of 5 frequency bands combined using a k-fold cross-validation technique. After several iterations, we observed that the models performed better at k = 5 in k-fold cross-validation. The models attained a good cross-validation score of 0.85, 0.9, 0.9, 0.9, and 0.85 for DT, EBDT, GB, RF, and SC, respectively, for the data subset comprising the features extracted from the augmented frequency bands. However, in the real-time examination, more than k-fold cross-validation, subject-wise validation creates acceptance. Later, the work was also evaluated under a 6-fold subject-wise cross-validation technique considering five subjects for training and one subject for testing and results in average cross-validation scores of 0.8 for DT, 0.91 for EBDT, 0.91 for GB, 0.85 for RF, and 0.81 for SC. Considering both validation strategies, the proposed method shows a more remarkable performance.

## Discussion

From the analysis shown in Fig. 10a) we can identify that when analyzing the graphical representation of combined frequency bands, all ML models utilized in the work are best suited for the automatic detection of insomnia where DT exhibits less. It also shows the comparative analysis of all frequency bands, which shows that the Gamma frequency band is the least suited for insomnia detection. For an accurate diagnosis, all frequency bands should be utilized for insomnia detection because each frequency band contributes to developing the sleep pattern. In addition, all types of insomnia variants are diagnosed when using all sleep frequency bands. The proposed model was validated using a 5-fold cross-validation technique to determine the efficiency of the model, which gives greater accuracy. The accuracy of the cross-validation technique for the GB model is 0.91, and that for EBDT is 0.90, which seems to be a comparatively good result. However, real-world scenarios of clinical disease detection practices validate the model using the subject-based approach. From Fig. 10b), it is clear that combining both layer 1 and layer 2 augmentation in the insomnia detection model provides comparatively better accuracy than using only one layer of augmentation. Each layer of DA is significant in developing the proposed detection model.

Advantages of Proposed Work:

- Combining CSA with CTGAN produces good accuracy.
- Employing double-layered augmentation gives better data security.
- Immensely practical for small datasets.
- Same data subs-sets size due to augmentation irrespective of sleep stage classification. Uniformity can be maintained while generating the subsets in different frequency bands.
- Fully automated without the involvement of human resources.
- Increases mobility and reduces discomfort.
- Perfectly suited for a comfortable real-time sleep examination.

Limitations

- Difficulty in reconstructing the original signal due to augmentation.

### Comparison of proposed work with state-of-the-art techniques

The proposed double-layered augmentation model has been compared with the existing works and is described in Table 10. The findings briefed in Table 10 reveal that compared to existing literature that relies on various methods, including sleep stage analysis, segmentation, etc., for generating training data in the automated detection of insomnia, the proposed work was found advantageous in many aspects. The spectral power of the EEG frequency band played a role in insomnia detection^17^ uses the idea of comparing the significant differences in spectral analysis of frequency bands for the detection of insomnia by analyzing the spectral power for healthy and insomniacs with hypnotic and nonhypnotic use and formulates that the beta and sigma frequency bands have high spectral power for insomnia groups with hypnotic use. Another study regarding spectral analysis utilizing Welch’s PSD estimates computed in insomnia and healthy subjects shows an elevated normalized power in deep sleep delta frequency for insomnia^48^. The analysis performed using spectral power did not result in automated detection or classification. Later, the PSG recordings in public databases helped researchers develop an automated model using machine learning or deep learning models to identify insomnia. However, the method implies a semiautomated detection system using sleep scoring, hence analyzing sleep stages and generating a hypothesis from the annotations recorded by a trained technician during sleep recording. In the semiautomated detection models, the EEG, ECG, EOG, or EMG signals are segmented into 30s epochs and classified sleep stages based on the sleep annotations. In the case of EEG signals used for detection, segmentation followed by EEG frequency bands on decomposition into sub-bands develop a model by examining subjects’ behavior in different sleep stages and found the REM stage is prominent in classification with a greater accuracy of 97.8%^18^. The main primitive behind the utilization of machine learning and deep learning models for classification is the use of a good amount of training data, which has been found very difficult to collect in the study of the development of insomnia detection models as it requires the whole night recording of the subject. Lack of sleep recording data in developing detection models for insomnia found the disorder to be less noticed by researchers. Apart from the five sleep stages extracted from sleep annotations according to AASM guidelines, Manish Sharma^19^ contributed a new practice of employing a combination of different sleep stages by designing a wavelet filter bank with minimal duration bandwidth localization and double half-band property for wavelet decomposition that forms eight subsets in developing the training data for the classification model achieves an accuracy of about 95% in N3 stage for both unbalanced data and balanced data but 84% using standard Daubechies wavelet decomposition. The difficulty of using real-time datasets has been overcome, and a detection model^49^ with a classification accuracy of 83% using various sleep stages has been developed. The detection models are generally implemented only using the same data for training and testing. But in^50^, they implemented the detection models in interpatient as well as in an intra-patient manner in identifying the behavior of detection models using 1D-CNN, which attains a high accuracy of 99% in SWS epochs, 98% for REM epochs for intra-patient paradigm and 87% high accuracy in REM epoch, 83% in SWS epochs that shows a better performance in the intra-patient model. A fully automated insomnia detection model was developed in^51^ that exhibits insomnia to be detected in the first 27.5 min that used RCMSE analysis for short-time detection using a Support Vector Machine (SVM) classifier generated an accuracy of 89.31 ± 6.04 found dominant in all detection models because of time consumption. The main disadvantage of the RCMSE analysis is that it cannot classify all insomnia variants, like early maintenance insomnia, that can only occur due to early awakening from sleep. A fully automated domain adaption method using three different datasets is adopted for the insomnia detection module, which uses a deep learning network to capture temporal representations and produces a greater accuracy of 90.9%, as described in^52^. The main focus of the proposed work is to modify the structure of conventional insomnia detection methods using machine learning or deep learning models employing sleep stage classification. The existing studies created training data subsets using sleep stage analysis, where the accuracy of various sleep stages is evaluated and compared. The deviation in the sleep stage creates variability in training data subset size for classification in machine learning or deep learning algorithms. The number of epochs in each sleep stage for the CAP sleep dataset from the Physionet database differs as 445 wake epochs for healthy whereas 3148 wake epochs for insomniacs. Similar variation can be visible in the remaining stages, as 280 N1 epochs are healthy against 170 for insomniacs. Likewise, epochs of 2172 N2, 1757 N3, and 1409 REM stages were observed in healthy subjects. Meanwhile, the N2, N3, and REM stages show an epoch count of 2241, 997, and 946 for insomniacs. The data subsets for the training data were created depending on the epochs in each sleep stage, and it was found to be non-uniform. Some data must be discarded to maintain uniformity in the given observations, which creates data loss. This practice of data loss can be refrained by using augmentation techniques for analysis instead of sleep stage segmentation. Data augmentation in the proposed study provides balanced data with minimal redundancy, offering better performance in classification algorithms. The presented double-layered augmentation approach creates data subsets by considering augmented frequency bands that provide balanced data in both categories. Moreover, in real-time implementation, the augmented approach does not need sleep stages annotated during recording, which may create data privacy for the subjects undergoing sleep examinations. Also, the augmented approach gains remarkable classification performance using various machine learning algorithms.

**Table 10:** Comparison of the existing state-of-the-art techniques with the proposed approach.

Reference	Signal	Channel	Dataset	Method	Accuracy	Remarks
Sharma et al.^25^	EEG	C4-A1	CAP-Insomnia: 9 Healthy:9	Sleep stage segmentation, Wavelet scattering network, classification using Ensemble bagged tree (EBT), Weighted K-nearest neighbor (WKNN), Trilayered neural network (TNN)	EBT: 92.90 WKNN:94.00 TNN:94.90	Used sleep stage annotations and hence unbalanced training data
Kumar et al.^26^	ECG	ECG1-ECG2	CAP	Extraction of ECG scalogram using CWT, CNN	98.91	Computationally intensive; need to convert ECG into scalogram
Kang et al.^17^	EEG		SHHS, No Ins:1386 Ins & no hypnotic use: 401 Hypnotic use with no ins:133 Hypnotic use with ins:65	EEG spectral power	-	Not automated. The sample size in each group is unbalanced.
Sharma et al.^18^	ECG	ECG1-ECG2	CAP Insomnia:7 Healthy: 6	Segmentation into 30s epochs, wavelet decomposition, norm feature extraction, classification (KNN, SVM, EBT) based on sleep stages	94	Utilized sleep stages and hence unbalanced training data.
Sharma et al.^19^	EEG	C4-A1	CAP Insomnia:7 Healthy:6	Sleep stage segmentation, subset creation from existing sleep stage, Wavelet decomposition, norm feature extraction, classification (EBDT) based on sleep stages	92	Sleep stage segmentation
Kusmakar et al.^23^	Actigraphy	-	Private data. Subjects recruited for Project	labeling each night as good/bad. Feature extraction. Classification (SVM, RF), Leave-one-out cross-validation	RF:80 SVM:75	Thresholding technique for data enhancement. Data recorded for 4 nights
Angelova et al.^24^	Actigraphy	-	Public data Insomnia: 26 Healthy:21	Thresholding (Th), Feature extraction, k-fold cross-validation, classification (SVM, RF)	SVM: 63 RF: 69 SVM & Th: 73 RF & Th: 84	Set up a wake-sensitivity threshold without any specific recommendations.
Yang et al.^50^	EEG	C4-A1	CAP Insomnia:8 Healthy:9	4 data subsets according to sleep stage annotations, BPF, Resampling, 1D-CNN, and 10-fold cross-validation. Evaluated inter and intra-patient paradigm	86.82 ± 5.43	Utilization of sleep stage annotations and hence unbalanced training data.
Kuo et al.^51^	EOG	EOG	Public data	BPF, segmentation into 30s epochs, MSE, and RCMSE feature extraction, classification (SVM, LDA, Ensemble of random subspace discriminant analysis)	LDA:86.50 ± 8.22 SVM:89.31 ± 6.04 Ensemble:88.69 ± 7.88	Utilization of manual sleep scoring
Proposed approach	EEG	C4-A1	CAP Insomnia:6 Healthy:6	Wavelet decomposition, signal level augmentation, feature extraction (entropy, spectral power, statistical), CTGAN, Classification (DT, EBDT, GB, RF, SC)	94	Completely automated, balanced training data development on different frequency bands using synthetic signal generation and augmentation on underlying signal characteristic, ensures data privacy.

## Conclusion

In the proposed approach, conventional and modern data augmentation methods are leveraged to overcome the limitations of existing detection models using single-channel EEG signals. The lack of physiological signals in the study of insomnia detection causes the researchers to give less attention to developing an automated model for detection. Conventional signal augmentation generates new synthetic signals, whereas a modern modified GAN architecture for augmenting the raw tabular data creates artificial data to be fed to train the classification model. Performing the model in a clinical environment, with the availability of multiple augmentations, provides an added advantage of data security for the subjects participating in the study. A maximum accuracy of 94% is obtained using RF in constructing the model for whole night EEG data, constituting all frequency bands; it also acquires a higher accuracy of 91% when validating the model using subject-wise cross-validation, the method primarily practiced in real-time examination. Previous detection models mainly used sleep annotations to analyze various sleep stages of the subjects, giving a way to analyze the EEG data in small intervals of time; there exists a possibility of omitting some data to maintain the uniformity of data subset size, which cannot be observed in the proposed approach which is having same subset size in all frequency bands avoiding data loss. However, the method of using sleep annotations, which are expected to change every 30 s, can be less accurate due to human involvement, and it cannot judge the sleep stage accurately. However, in real-time applications, these guidelines may be violated due to the intrusion of one sleep stage into another, making the detection model based on sleep stages less explainable for real-time diagnostic purposes. Considering the frequency bands of the EEG signal corresponding to each sleep pattern that changes abruptly whenever a spike occurs, benefiting the utilization of frequency bands in developing the insomnia detection model more accurately for diagnosis. The approach used a single-channel EEG signal to analyze the study, which increases mobility during the examination and reduces the discomfort that disturbs sleep, which was very well experienced using multi-channel PSG signals. It is less expensive than extracting PSG signals, and it also requires a simple experimental setup for real-time study. The proposed method analyzes sleep EEG in a novel way by augmenting its frequency bands in different forms, enabling a fully automated sleep-scoring independent detection, immensely effective for small datasets, and can be ideally suited for real-time sleep examination using wearable devices constituting recording in a comfortable sleep environment.

## Data Availability

Data utilized in the work is publicly available on the Physionet website https://archive.physionet.org/cgi-bin/atm/ATM.
